# What is the role of CD4 count in a large public health antiretroviral programme?

**DOI:** 10.4102/sajhivmed.v17i1.446

**Published:** 2016-02-12

**Authors:** Michelle Moorhouse, Francesca Conradie, Francois Venter

**Affiliations:** 1 Wits Reproductive Health and HIV Institute, Johannesburg, South Africa; 2 Right to Care and Clinical HIV Research Unit, University of the Witwatersrand, South Africa

HIV clinicians have relied on the CD4 count for more than three decades as being central to key issues in managing HIV-positive patients. The CD4 count is an important predictor of disease progression^1,2,3,4,5^ and death,^6,7,8^ and has informed us when to start antiretroviral therapy (ART), opportunistic infection (OI) risk stratification (especially in late presenting patients), when to start and stop OI prophylaxis or management, as well as in monitoring response to treatment.^8,9,10^ However, in areas where viral loads are readily available and patients are virologically suppressed and stable, the question arises – is there a role for continued CD4 count monitoring in this setting?

This question merits consideration. South Africa bears 20% of the global HIV burden, with approximately 3 million people accessing ARVs and a similar number still in need of ART. With such large numbers of people accessing care through a public health programme, cost remains a critical factor that needs to be considered with regard to each and every aspect of the programme. Savings that may seem miniscule considered on an individual basis, are not inconsiderable when scaled up to the size of South Africa’s ARV programme.

The South African Department of Health (DoH) guidelines, which are evidence-based, have traditionally followed the World Health Organization (WHO) recommendations. The first DoH guidelines, in 2004, recommended treatment initiation at CD4 count < 200 cells/mm^3^. In 2009, the CD4 threshold for treatment was increased to 350 cells/mm^3^. The Haiti study, published in NEJM in 2010, was a randomised, controlled trial that demonstrated reduced mortality and incident tuberculosis (TB) in patients starting ART at a CD4 count threshold of < 350 cells/mm^3^ (compared with patients waiting to commence therapy at a threshold of < 200 cells/mm^3^).^11^

The previous WHO recommendation of treatment initiation at CD4 counts < 500 cells/mm^3^ was adopted into the South African DoH guidelines in January 2015. However, the evidence supporting the 500 cells/mm^3^ CD4 threshold has been less clear because, until recently, no randomised, controlled trial (RCT) had definitively shown any benefit in initiating ART at a CD4 count higher than 350 cells/mm^3^. Data from observational studies suggested reduced morbidity (especially from non-HIV-related events) and mortality with ART initiation at higher CD4 counts. The TEMPRANO and START studies are RCTs investigating optimal CD4 count initiation thresholds. Data from TEMPRANO are more difficult to interpret, due to the study methodology that, in two of the four study arms, shifted CD4 initiation thresholds several times. The reason for this was that national guidelines in the countries in which TEMPRANO was conducted changed according the WHO guideline recommendations.^12^

HPTN 052 is an RCT that showed reduced morbidity, but not mortality,**** associated with starting ART at a CD4 count of 350 cells/mm^3^ – 550 cells/mm^3^ (compared with < 250 cells/mm^3^). Despite the fact that mortality was not reduced in HPTN 052, the major benefit demonstrated in the study was a 96% reduction in HIV infection amongst serodiscordant couples in which the HIV-positive partner was initiated at the higher CD4 count threshold, as opposed to delaying to CD4 < 250 cells/mm^3^.^13^ These data are a compelling argument for moving towards ‘Test and Treat’ (ART initiation independent of CD4 count), as recommended in the latest iteration of WHO guidelines (2015).^14^

The START study, an RCT, compared early ART initiation in asymptomatic patients with CD4 counts > 500 cells/mm^3^ to deferred initiation at CD4 count ≤ 350 cells/mm^3^. The study commenced in 2009 and enrolled over 4000 participants. The START study was stopped prematurely, in 2015, and a press release was issued by the National Institute of Allergy and Infectious Diseases based on the recommendations of the study’s independent Data and Safety Monitoring Board (DSMB). Following their review of interim study data, the DSMB found that the risk of developing serious illness or death in the early treatment arm was reduced by 53%, compared to the results for those in the deferred treatment arm. START study, therefore, provides robust evidence that it is beneficial to patients to initiate ART irrespective of their CD4 count and whether or not they are asymptomatic.^15^ These findings have global implications, in that we now have proof of the double impact of early ART initiation: the benefit to the health of the HIV-infected individual as proven in START, as well the reduction of HIV transmission risk, as proven in HPTN052.

In its 2014–2015 HIV treatment guidelines, the DoH endorses the use of the viral load as the preferred test for monitoring response to ART, a shift that aligns with current WHO guidelines. The viral load detects virological failure before immunological or clinical failure become apparent and is one of the main reasons for the shift towards the viral load as the preferred test for monitoring response ART in HIV-infected patients.

One of the barriers to viral load monitoring is the cost. By reducing the overall cost of laboratory testing, for example by stopping routine CD4 count monitoring in virologically suppressed patients, will significantly increase the amount of money available which could be channeled to covering the costs of increasing numbers of viral loads as the size and cost of South Africa’s ARV programme increases as we strive for 90:90:90.

The evidence supports moving away from using CD4 counts as a means for monitoring ART. Data from RCTs and observational studies suggest that, once virological suppression is achieved, in the majority of patients, CD4 counts remain stable over time,^16^ and this conclusion is supported by cohort data from high HIV prevalence areas, namely South Africa and Uganda. The South African cohort showed that CD4 counts tend to be maintained over 200 cells/mm^3^ in more than 92% of patients to 10 years. Moreover, those who did experience CD4 decreases, these were self-limiting in more than 90% of cases^16^. The Ugandan cohort had similar findings, in that amongst virologically suppressed patients with CD4 counts ≥ 200 cells/mm^3^, less than 5% of patients experienced CD4 declines to < 200 cells/mm^3^; in those whose subsequent CD4 counts were measured, more than 80% were transient.^17^

Currently, waiting for a CD4 count to confirm eligibility for ART in patients may in fact be a barrier to treatment and delay ART initiation. Yet, we have seen, for example, in South Africa’s prevention of mother-to-child transmission (PMTCT) programme, that it is possible to initiate ART without waiting for a CD4 count result. The very test that may be the key criterion for treatment eligibility should not also, ironically, function as the barrier to treatment.

Until such times as South Africa moves towards ‘Test and Treat’, which is supported by START data, the main role of CD4 count monitoring will be in determining eligibility for ART, as well as OI prophylaxis or management, and assisting clinicians in deciding when OI prophylaxis/treatment should be discontinued, to avoid prolonged administration of drugs with not insubstantial side effect profiles and toxicity in otherwise healthy patients.

In the scenario of ‘Test and Treat’, CD4 count testing will still continue to play an important role in the baseline assessment of patients to inform initial clinical management decisions, particularly for those presenting late to care, as well as those on ART where clinical deterioration or virological failure is suspected or evident.

The Southern African HIV Clinicians Society is of the opinion that the CD4 count retains an important role in the care of HIV-infected patients. The Society also believes that the role of the CD4 count is changing, as new RCT evidence becomes available to guide optimal patient care that is balanced, out of necessity, against cost concerns in a public health programme of the magnitude of South Africa’s antiretroviral programme. Going forward, the optimal use of the CD4 count in South Africa’s programme would be to guide the initiation and discontinuation of OI prophylaxis/management and in assessing late presenting patients, or patients on ART when clinical or immunological failure is suspected. Once patients are initiated onto ART, we recommend that one further CD4 count be checked, at 6 months, to guide decisions regarding OI prophylaxis/management, and thereafter only if clinically indicated. In terms of monitoring the response to ART, the preferred test remains the viral load. In stable, virologically suppressed patients, the CD4 count offers little value and contributes significantly to costs in an ARV programme as large as that found in South Africa.
